# A Nonsense Variant in *COL6A1* in Landseer Dogs with Muscular Dystrophy

**DOI:** 10.1534/g3.115.021923

**Published:** 2015-10-01

**Authors:** Frank Steffen, Thomas Bilzer, Jan Brands, Lorenzo Golini, Vidhya Jagannathan, Michaela Wiedmer, Michaela Drögemüller, Cord Drögemüller, Tosso Leeb

**Affiliations:** * Neurology Service, Department of Small Animals, Vetsuisse Faculty, University of Zurich, Winterthurerstrasse 260, 8057 Zurich, Switzerland; † Institute of Neuropathology, University Hospital Düsseldorf, Moorenstraße 5, 40225 Düsseldorf, Germany; ‡ Institute of Genetics, Vetsuisse Faculty, University of Bern, 3001 Bern, Switzerland

**Keywords:** Mendelian traits, animal model, dog, veterinary genetics, whole-genome sequencing

## Abstract

A novel canine muscular dystrophy in Landseer dogs was observed. We had access to five affected dogs from two litters. The clinical signs started at a few weeks of age, and the severe progressive muscle weakness led to euthanasia between 5 and 15 months of age. The pedigrees of the affected dogs suggested a monogenic autosomal-recessive inheritance of the trait. Linkage and homozygosity mapping indicated two potential genome segments for the causative variant on chromosomes 10 and 31 harboring a total of 4.8 Mb of DNA or 0.2% of the canine genome. Using the Illumina sequencing technology, we obtained a whole-genome sequence from one affected Landseer. Variants were called with respect to the dog reference genome and compared with the genetic variants of 170 control dogs from other breeds. The affected Landseer dog was homozygous for a single, private nonsynonymous variant in the critical intervals, a nonsense variant in the *COL6A1* gene (Chr31:39,303,964G>T; *COL6A1:c.289G>T*; p.E97*). Genotypes at this variant showed perfect concordance with the muscular dystrophy phenotype in all five cases and more than 1000 control dogs. Variants in the human *COL6A1* gene cause Bethlem myopathy or Ullrich congenital muscular dystrophy. We therefore conclude that the identified canine *COL6A1* variant is most likely causative for the observed muscular dystrophy in Landseer dogs. On the basis of the nature of the genetic variant in Landseer dogs and their severe clinical phenotype these dogs represent a model for human Ullrich congenital muscular dystrophy.

Muscular dystrophies are a heterogeneous group of inherited, degenerative disorders characterized by progressive muscular dysfunction (Flanigan 2012). Duchenne muscular dystrophy caused by variants in the X-chromosomal *DMD* gene and a complete loss of function of dystrophin is the textbook representative for these entities (Koenig *et al.* 1987). However, although many of the muscular dystrophies are caused by single-gene defects, there is considerable variance in the phenotype (age of onset, severity and localization of clinical symptoms, rate of progression, etc.). This complexity arises from the fact that many genes are required for the structural integrity and proper function of skeletal muscle and that different genetic variants within a given gene can lead to quite distinct phenotypes. This again can be seen with the *DMD* gene: variants that lead to a complete loss of function cause the severe Duchenne muscular dystrophy, whereas variants that only partially affect dystrophin function result in the milder Becker muscular dystrophy (Hart *et al.* 1987; Koenig *et al.* 1989). Dystrophin interacts with many other skeletal muscle proteins. Variants in genes encoding these so-called dystrophin-associated proteins can lead to very similar phenotypes as variants in the *DMD* gene itself.

Another group of muscular dystrophies with a much less well understood pathophysiology are the collagen VI myopathies. Collagen VI is a ubiquitously expressed extracellular matrix protein, composed of two chains of 140−150 kDa each, named alpha1(VI) and alpha2(VI), and one larger chain of 240−300 kDa named alpha3(VI) (Furthmayr *et al.* 1983). The three chains fold together into a triple-helical collagen VI molecule (monomer) that further assembles into dimers and tetramers. The tetramers are secreted into the extracellular space where they form collagen VI beaded microfibrils. In muscle, the microfibrillar network of collagen VI surrounds the basement membrane of fibers, binding components of the extracellular matrix and transferring mechanical and biochemical signals from the extracellular matrix to the muscle cell (De Palma *et al.* 2014). The three subunits of collagen VI are encoded by the *COL6A1*, *COL6A2*, and *COL6A3* genes. Variants in any of these genes can cause the severe Ullrich muscular dystrophy or the much milder Bethlem myopathy (Jöbsis *et al.* 1996; Camacho Vanegas *et al.* 2001). Many different variants have been identified in human patients, and it is becoming increasingly clear that the phenotypic spectrum of these collagen VI myopathies is almost continuous depending on the specific genetic variants (Briñas *et al.* 2010). A complete list of all known functional human variants in the collagen VI genes is compiled at the Leiden Muscular Dystrophy pages (http://www.dmd.nl/).

Dogs have been recognized as valuable models for many human hereditary diseases, and the Online Mendelian Inheritance in Animals database currently lists ∼350 potential canine models for human diseases. In roughly 200 of these, the causative genetic defect is known (Nicholas and Hobbs 2014; http://omia.angis.org.au/home/). Different muscular dystrophies also have been described in dogs. Golden Retrievers with a Duchenne-like muscular dystrophy caused by a *DMD* variant are an extremely valuable animal model because *Dmd*-mutant mice do not recapitulate the severe human (and canine) phenotypes (Cooper *et al.* 1988; Valentine *et al.* 1988, 1992). Many other cases of muscular dystrophies in dogs and other companion animals have been published. Recently, the case of a myopathic Labrador Retriever was reported that showed greatly reduced or absent sarcolemmal collagen VI expression (Marioni-Henry *et al.* 2014). Collagen VI expression in this dog was still detectable by antibody staining in the endomysium. The clinical phenotype of this Labrador Retriever resembled an intermediate severity compared with human Bethlem myopathy or Ullrich congenital muscular dystrophy. Because the genetic defect in this Labrador Retriever is not known, it remains to be determined whether this represents a primary collagen VI defect or whether the reduction in collagen VI is a secondary effect of some other genetic lesion. Therefore, without the conclusive identification of the underlying causative genetic defect, the value of these reports for our basic understanding of muscle function and a comparative analysis with respect to the human diseases remains limited.

In this report, we describe a novel canine muscular dystrophy that occurred in Landseer dogs. We also provide an initial limited characterization of the clinical and histopathologic phenotype. The main focus of this study is on the identification of the presumed causative genetic defect by a positional cloning approach in combination with whole-genome sequencing of an affected dog.

## Materials and Methods

### Ethics statement

All animal experiments were performed according to the local regulations. The dogs in this study were examined with the consent of their owners. The collection of blood samples was approved by the “Cantonal Committee For Animal Experiments” (Canton of Bern; permit 23/10). The clinical examinations of affected Landseer dogs and their littermates including the collection of muscle biopsies was performed in the course of standard veterinary diagnostics, with the consent and on behalf of the dogs’ owners. The clinical examinations therefore did not constitute an animal experiment in the legal sense and did not require ethical approval.

### Clinical examinations

Three affected Landseer siblings were brought to the neurology service at the Vetsuisse Faculty of Zurich for examination and diagnostic testing. A history of all dogs was collected from the breeder. All dogs of the litter underwent both a general physical examination and a detailed neurologic examination, as well as having complete blood counts, serum biochemistry panels and urinalysis. Electromyography and motor nerve conduction velocity were performed on an electrodiagnostic device (Neuroscreen, Toennies AG, Germany) with concentric and monoaxial needle electrodes while the animals were maintained under general gaseous anesthesia. For motor nerve conduction velocity, the tibial nerve was examined along its entire course by the use of stimulation sites at the trochanteric fossa, the popliteal fossa, with recording at the interosseous muscle. Muscle biopsies from *Musculus triceps*, *Musculus biceps femoris*, and *Musculus tibialis cranialis* were collected after electrodiagnostic testing with an open standard surgical approach. Biopsies were placed in a container without any additives and were submitted for histopathologic assessment by overnight express to the Institute of Neuropathology of the Heinrich-Heine-University, Düsseldorf (Germany). Clinical data on two affected Landseer dogs from a second family was kindly contributed by their owners and the attending veterinarians.

### Histopathologic examinations

Muscle biopsies were frozen at −135° in isopentan and stored at −80° for further analysis. The frozen biopsies were cut using a cryosat (CM 1900, Leica Biosystems, Wetzlar, Germany) into sections of 7-µm thickness and stained with hematoxylin and eosin, modified Gomori trichrome (Engel), and acidic phosphatase. All sections were examined under a transmitted light microscope (Orthoplan; Leica Microsystems) in 100×, 400×, and 1000× magnification. Additional cryosections were used for immunohistochemic stainings using an antibody against the rod domain of dystrophin (1:100, NCL-DYS1; Novocastra Laboratory).

### Animal selection for the genetic analysis

This study describes findings from two Landseer families. The first family (Figure 2A) consisted of the parents and six offspring that were available for ethylenediaminetetraacetic acid blood sample collection. A seventh offspring was affected by muscular dystrophy but was euthanized before samples could be taken. From the second family, we had access to the blood samples of two affected female offspring. The sample of the second affected dog from this family became available only after we had already completed the initial genetic mapping of the disease locus. Thus, in total we had samples from five affected Landseer dogs, three nonaffected full-siblings, and two nonaffected parents.

We additionally had samples from 48 other Landseer dogs without known relationships to the two families that had been donated to the biobank of the Institute of Genetics. Finally, we also used 404 samples from Newfoundland dogs and 473 samples from 63 various diverse dog breeds, which are listed in detail in supporting information, Table S1.

### DNA samples and genotyping

We isolated genomic DNA from EDTA blood samples with the Nucleon Bacc2 kit (GE Healthcare). Genotyping was done on Illumina CanineHD chips containing 173,662 SNPs by GeneSeek/Neogen. Genotypes were stored in a BC/Gene database version 3.5 (BC/Platforms).

### Linkage and homozygosity mapping

For the linkage analysis we had Illumina CanineHD SNP chip genotypes from a single family consisting of eight animals in total. This family comprised both nonaffected parents and six offspring, of which three were affected. We used the Merlin software (Abecasis *et al.* 2002) and a fully penetrant, recessive model of inheritance to analyze the data for parametric linkage.

We used PLINK v1.07 (Purcell *et al.* 2007) to search for extended intervals of homozygosity with shared alleles as described (Drögemüller *et al.* 2009). The final critical intervals were defined by visual inspection of all SNP chip genotypes of the four genotyped cases on chromosomes 10 and 31 in an Excel-file.

### Gene analysis

We used the dog CanFam 3.1 assembly for all analyses. All numbering within the canine *COL6A1* gene corresponds to the accessions XM_003434001.3 (mRNA) and XP_003434049.2 (protein).

### Whole-genome sequencing of an affected Landseer dog

We prepared a polymerase chain reaction (PCR)-free fragment library with 300-bp insert size and collected 330,331,465 Illumina HiSeq2500 paired-end reads (2 × 100 bp) or roughly 14.2× coverage. We mapped the reads to the dog reference genome using the Burrows-Wheeler Aligner version 0.5.9-r16 (Li and Durbin 2009) with default settings and obtained 380,485,021 uniquely mapping reads. After sorting the mapped reads by the coordinates of the sequence with Picard tools, we labeled the PCR duplicates also with Picard tools (http://sourceforge.net/projects/picard/). We used the Genome Analysis Tool Kit [GATK version v2.3-6, (McKenna *et al.* 2010)] to perform local realignment and to produce a cleaned BAM file. Variant calls were then made with the unified genotyper module of GATK. Variant data for each sample were obtained in variant call format (version 4.0) as raw calls for all samples and sites flagged using the variant filtration module of GATK. Variant calls that failed to pass the following filters were labeled accordingly in the call set: (i) Hard to Validate MQ0 ≥ 4 & ((MQ0 / (1.0 * DP))>0.1); (ii) strand bias (low quality scores) QUAL<30.0 || (Quality by depth) QD<5.0 || (homopolymer runs) HRun>5 || (strand bias) SB>0.00; (iii) SNP cluster window size 10. The snpEFF software (Cingolani *et al.* 2012) together with the CanFam 3.1 annotation was used to predict the functional effects of detected variants. We considered the following snpEFF categories of variants as non-synonymous: NON_SYNONYMOUS_CODING, CODON_DELETION, CODON_INSERTION, CODON_CHANGE_PLUS_CODON_DELETION, CODON_CHANGE_PLUS_CODON_INSERTION, FRAME_SHIFT, EXON_DELETED, START_GAINED, START_LOST, STOP_GAINED, STOP_LOST, SPLICE_SITE_ACCEPTOR, SPLICE_SITE_DONOR. The critical intervals contained 4,788,534 total and 50,649 coding nucleotides, respectively. In our resequencing data, we had ≥4× coverage on 4,671,977 bp of the critical interval (97.6%) and on 49,327 (97.4%) of the coding bases.

### Sanger sequencing

We used Sanger sequencing to confirm the Illumina sequencing results and to perform targeted genotyping for selected variants. For these experiments, we amplified PCR products using AmpliTaqGold360Mastermix (Applied Biosystems). PCR products were directly sequenced on an ABI 3730 capillary sequencer (Applied Biosystems) after treatment with exonuclease I and shrimp alkaline phosphatase. We analyzed the Sanger sequence data with Sequencher 5.1 (GeneCodes).

### Data availability

File S1 illustrates the clinical phenotype of an affected Landseer dog. Table S1 contains the coordinates of the linked genome intervals. Table S2 contains the coordinates of the homozygous intervals. The raw SNP chip genotypes are available upon request. The genome sequencing data were deposited in the European Nucleotide Archive under accession PRJEB9437. Table S3 lists the breeds of all control dogs.

## Results

### Clinical presentation and laboratory findings

We investigated a litter of seven Landseer dogs. Two male and two female puppies were affected by progressive muscle weakness (File S1). One male puppy had been euthanized because of severe clinical signs in the first weeks of life without any ancillary diagnostic tests or necropsy. We examined the remaining three affected littermates, which were presented because of reluctance to move and prolonged sleeping episodes compared to their three normal siblings during the first few weeks of life.

The male and the two female cases were clinically and neurologically examined at 14 and 18 weeks of age. No abnormalities were found on general physical examination of the male dog. On neurologic evaluation, the affected male dog was mentally alert but only able to ambulate for a few steps before lying down. He had a short, strided gait in the thoracic and pelvic limbs and while standing his back was arched and the pelvic limbs were placed under the abdomen. Stifle and tarsal joints were kept in a slightly flexed position. On palpation, the muscles of the limbs seemed to be atrophied and the articulations of elbow, carpus, hip, and tarsus had a decreased range of motion. Manipulation of the joints seemed to be painful for the dog. Spinal reflexes and proprioceptive testing was normal, as was the remainder of the neurologic examination. Both female dogs were examined and were found to be affected in different degrees of severity. Although the clinical and neurologic status of one female dog was comparable with that of the male dog, the second female was nonambulatory and had difficulties with swallowing, regurgitation, and salivation. This dog had an elevated body temperature of 39.6° and auscultation of the lungs revealed harsh sounds. Neurologically, the gag reflex was reduced but no other cranial nerve abnormalities were detected. Compared with her littermates, the muscles of this dog were more profoundly atrophied. The remainder of the neurologic tests did not differ from those of her littermates.

In all affected dogs the creatine kinase activity was elevated (422, 935, 1149 IU/L; reference values: 51-191 IU/L). Hematology of the more severely affected female revealed an elevated leukocyte cell count, whereas it was normal in the other dogs. Urinalysis was normal in all dogs. Serology for *Neospora canis* and *Toxoplasma gondii* was tested in two dogs, and results came back negative.

Thoracic radiographs revealed generalized megaesophagus and aspiration pneumonia in the severely affected female. In the other two dogs, radiographs did not show evidence of esophageal, lung, or cardiac pathology. Radiographic examination of the hips, stifle, elbow, and carpal articulation did not reveal any degenerative findings or conformational abnormalities.

We then performed electromyography to measure the nerve conduction velocity. In all three affected dogs, appendicular and axial skeletal muscles were examined. There was a dense pattern of fibrillation potentials and complex repetitive discharges in all tested myotomes. The nerve conduction velocity in the tibial nerves was within normal limits along the entire course of the nerve.

All three affected dogs were euthanized at 5 months of age after histologic diagnosis and because of the severity of their clinical signs. The three clinically unaffected littermates were examined by means of a physical and neurologic examination and routine hematology and biochemical profile. No clinical abnormalities were detected, and results of laboratory testing, including levels of creatine kinase, were normal. The dam and one unaffected female sibling were screened for subclinical evidence of the disease by EMG examination and muscular biopsy with both tests yielding negative results.

Similar clinical findings were reported by the owners and their veterinarians in another litter of Landseer dogs with two affected siblings (File S1). These dogs were euthanized at 8 and 15 months of age because of severe progressive muscle wasting.

### Pathologic findings

**Figure 1:**
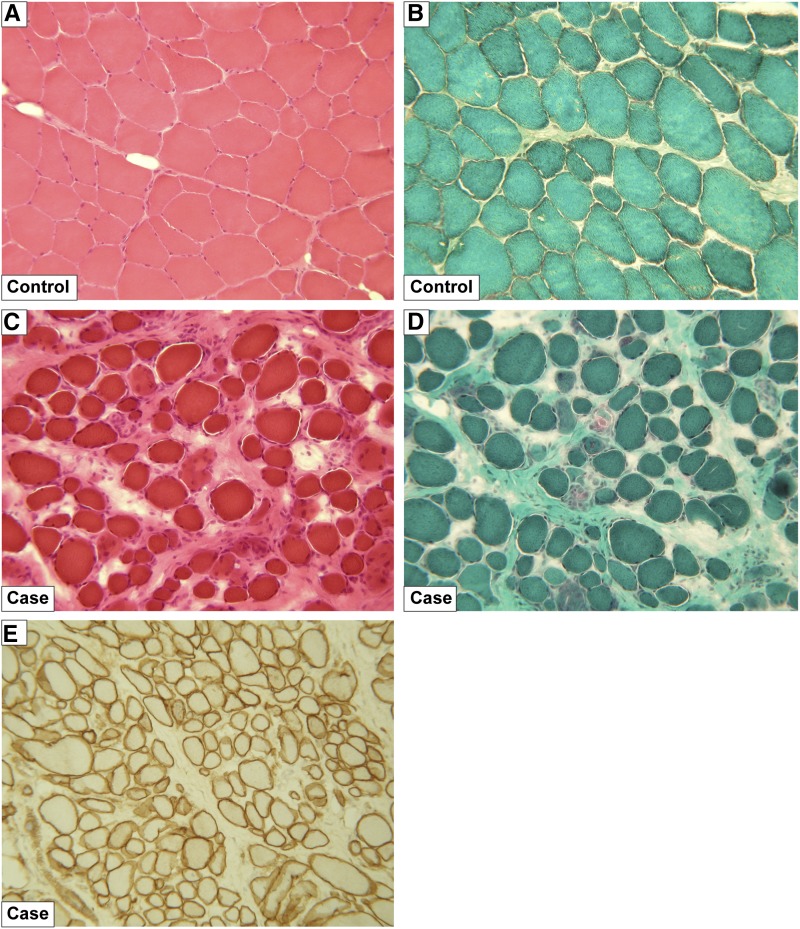
Skeletal muscle pathology. (A) *M. quadriceps* of a 4-months-old healthy Landseer dog with normal muscle texture without signs of significant pathologic changes. Especially, muscle and fiber size configuration and variation, sarcolemma, nuclear structure and distribution as well as endomysial connective tissue are of physiological appearance. hematoxylin and eosin (HE) staining, 100×. (B) Same muscle in the Gomori-Trichrome staining according to Engel. Normal muscle tissue and fiber structure without considerable pathologic changes are obvious, especially intrasarcoplasmal texture, mitochondria, muscular nuclei, and endomysial connective tissue are unobtrusive. (C) *M. quadriceps* of an affected full-sibling of the control dog shown above. HE staining, 100×. The muscle texture is severely disturbed showing a pathologic muscle fiber variation in size and configuration, signs of de- and regeneration, *e.g.*, nuclear proliferation and distribution, as well as a remarkable increase and distortion of the endomysial connective tissue. (D) Same muscle in the Gomori-Trichrome staining according to Engel points up the pathologic muscle changes. Interestingly and in contrast to other forms of muscle dystrophy such as the Duchenne/Becker analogous disease in Golden Retrievers, the sarcoplasm structures and the mitochondria are not markedly altered. (E) Immunohistochemistry with an antibody against dystrophin performed on the same muscle as in (C) and (D). The diseased muscle shows no deficiency and regular expression of dystrophin. Thus this particular muscular dystrophy is markedly different from the Duchenne/Becker types of muscular dystrophy, where dystrophin expression is altered.

We investigated the histopathology of skeletal muscle samples. All affected dogs showed pathologic variation in muscle fiber size and most of the fibers were round to anisomorphic instead of having a (physiological) polygonal shape. Many small fibers were scattered throughout the biopsies, along with some hypercontracted fibers. Sporadically invading phagocytes grouped around degenerating fibers. Staining for acidic phosphatase showed an increase of activity, indicating degeneration to necrosis in a large number of fibers. Some biopsies correlated to an advanced stage of disease with distinct proliferation of the endomysial connective tissue and an increase of adipose tissue. These findings demonstrate different stages of muscular destruction with compensatory hypertrophy and replacement of lost fibers by connective tissue and fat cells. The histhopathologic findings were typical for a muscular dystrophy. However, relatively normal immunohistochemistry findings with an anti-dystrophin antibody clearly showed that the muscular dystrophy in the Landseer dogs is different from the Duchenne/Becker type. The unaffected littermates did not show any histopathologic alterations (Figure 1).

### Genetic mapping of the causative mutation

The pedigrees of the two Landseer families were consistent with a monogenic autosomal-recessive mode of inheritance. The two litters had a common great-great-grandmother in their maternal lineages. On their paternal ancestry we could not detect common ancestors within five generations.

At the time of the initial mapping of the causative locus we had samples from only four reliably diagnosed cases: Three affected littermates from the first family and one isolated case from the second family. We additionally had samples from the three nonaffected siblings and both nonaffected parents of the first family. We obtained 173,662 SNP chip genotypes for each of these nine dogs. We then performed a parametric linkage analysis using a fully penetrant, monogenic autosomal recessive model of inheritance in the family with its 10 informative meioses. We obtained positive LOD scores for five genome segments containing 74 Mb in total. The greatest LOD scores with 1.6 were obtained on chromosomes 24 and 31 (Table S1).

On the basis of the pedigree records and typical dog breeding practices, we hypothesized that all affected dogs most likely were inbred to one single founder animal. Under this scenario, the affected individuals were expected to be identical by descent for the causative mutation and flanking chromosomal segments. We therefore analyzed the four available cases for extended regions of homozygosity with simultaneous allele sharing and found 18 genome regions with a total of 67 Mb that fulfilled our homozygosity search criteria (Table S2).

**Figure 2:**
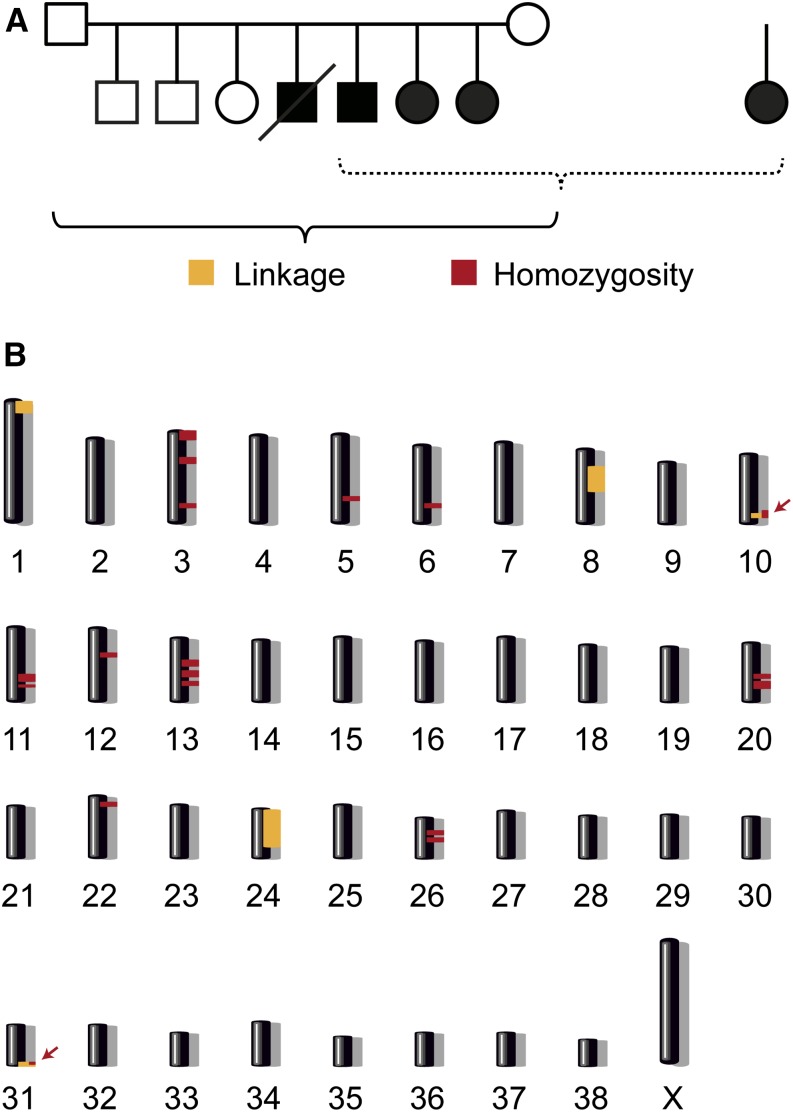
Mapping strategy. (A) A family comprising six offspring with their parents and one isolated case were available for the initial mapping of the disease locus. We performed parametric linkage analysis for a recessive trait in the family and homozygosity analysis across the four cases. (B) The analyses yielded five linked genome segments (orange) and 18 homozygous genome segments (red). Only two regions on chromosomes 10 and 31 showed both linkage and homozygosity and were considered the critical intervals (arrows). Specifically, these regions corresponded to Chr10:61,871,450-66,047,210 and Chr31:38,752,158-39,364,930.

When we intersected the linked intervals from the family and the homozygous intervals from the four cases, only two intervals on chromosomes 10 and 31 remained. Taken together these two intervals had a size of 4.8 Mb and were considered the minimal critical interval for the subsequent analyses (Figure 2).

### Mutation identification

To obtain a comprehensive overview of all variants in the critical intervals we resequenced the whole genome of one affected Landseer. We collected 330 million 2 × 100 bp paired-end reads corresponding to 14.2× coverage of the genome. We called SNPs and indel variants with respect to the reference genome of a presumably nonaffected Boxer. Across the entire genome, we detected ∼5.6 million variants of which ∼2.8 million were homozygous variants (Table 1). Within the critical intervals there were 10,190 variants, of which 38 were predicted to be nonsynonymous.

**Table 1:** Variants detected by whole-genome resequencing of an affected Landseer

Filtering Step	Number of Variants
Variants in the whole genome*^a^*	2,808,714
Variants in the critical intervals on CFA 10 and 31	10,190
Variants in the critical intervals that were absent from 170 other dog genomes	18
Nonsynonymous variants in the whole genome*^a^*	11,798
Nonsynonymous variants critical intervals on CFA 10 and 31	38
Nonsynonymous variants in the critical intervals that were absent from 170 other dog genomes	1

a The sequences were compared to the reference genome (CanFam 3.1) from a Boxer. Only variants that were homozygous in the affected Landseer are reported.

**Figure 3:**
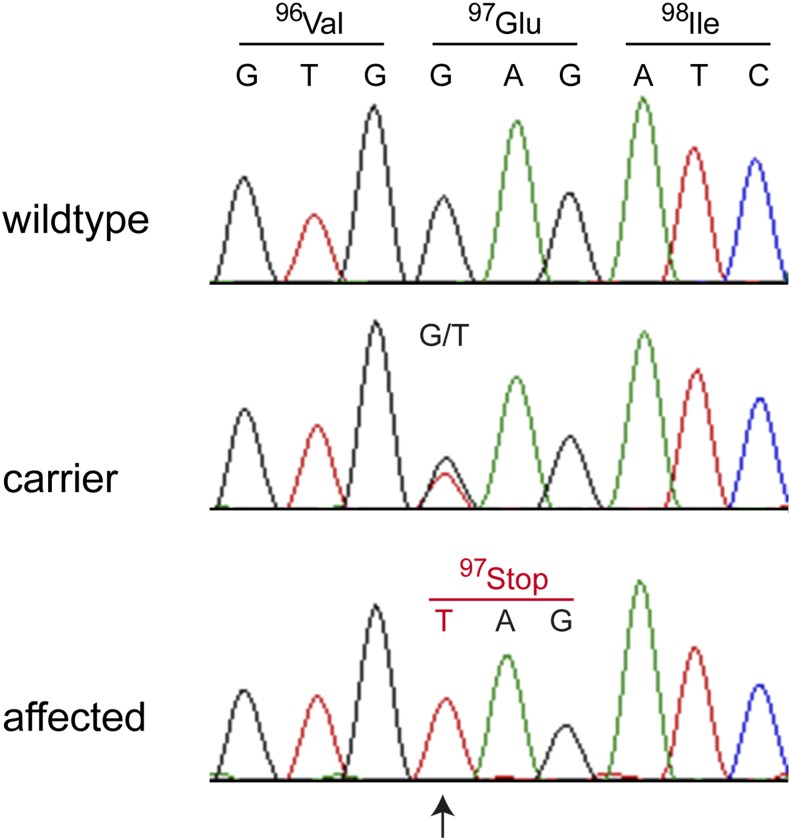
Electropherograms of the *COL6A1:c.289G>T* variant. A fragment harboring exon 3 and flanking sequences of the *COL6A1* gene was amplified by polymerase chain reaction and sequenced with the Sanger method. Shown are representative traces from Landseer dogs with the three different genotypes. The position of the variant is indicated by an arrow.

We further compared the genotypes of the affected Landseer with 170 dog genomes of various breeds that had been sequenced in the course of other ongoing studies. We hypothesized that the mutant allele at the causative variant should be completely absent from all other dog breeds in our sample set. After this filtering step only a single non-synonymous private variant remained in the critical intervals, Chr31:39,303,964G>T or *COL6A1:c.289G>T*. We confirmed this variant by Sanger sequencing (Figure 3) and genotyped it in 58 Landseer dogs, 404 Newfoundland dogs, and 473 dogs of 63 diverse other breeds. The genotypes showed perfect cosegregation with the muscular dystrophy in Landseer dogs and we did not find the variant allele outside of the Landseer breed (Table 2; Table S3). The *COL6A1:c.289G>T* variant represents a nonsense mutation in exon 3 of the *COL6A1* gene, encoding the alpha-1 chain of collagen type VI. The variant leads to an early premature stop codon and is predicted to truncate more than 90% from the *COL6A1* open reading frame (p.E97*).

**Table 2:** Association of the *COL6A1:c.289G>T* genotypes with muscular dystrophy

Genotype	Landseer Cases*^a^*	Landseer Controls*^b^*	Newfoundland Dogs*^c^*	Control Dogs From Other Breeds*^d^*
G/G	−	33	404	473
G/T	−	20	−	−
T/T	5	−	−	−

a Four cases had been available at the beginning of the project. A fifth case, full sister to the case from the second family, became available only after the genetic mapping of the locus had already been completed.

b The Landseer controls consisted of 5 close relatives of the affected dogs and 48 Landseer dogs without known relationships to the two families with muscular dystrophy. Of these 48 dogs, 33 were homozygous G/G and 15 were heterozygous G/T. In this small and not necessarily representative cohort, the carrier frequency was thus 31%.

c We genotyped a large number of Newfoundland Dogs as the Landseer breed was originally derived from Newfoundland Dogs. The 404 genotyped samples were collected in the course of other ongoing research projects.

d These 473 dogs were specifically genotyped for the *COL6A1:c.289G>T* variant. The genome sequences of 170 independent control dogs were also homozygous G/G at this variant. Therefore, the total number of control dogs adds up to 643.

## Discussion

In this study, we describe a novel canine muscular dystrophy in Landseer dogs. The clinical and histhopathologic findings were similar to the severe Duchenne muscular dystrophy in Golden Retrievers with a genetic defect in the X-linked *DMD* gene encoding dystrophin (Cooper *et al.* 1988; Valentine *et al.* 1988; Valentine *et al.* 1992). However, because male and female Landseer dogs were equally affected in the two studied families, an X-linked genetic defect seemed very unlikely. We therefore applied a positional cloning approach to elucidate the causative genetic defect. With only one complete family and one additional case, we were able to map the disease locus to just 0.2% of the dog genome. The increasing availability of “personal genomes” in canine genetics allowed us to quickly identify the only nonsynonymous genetic variant in the critical genome regions that was exclusively present in an affected Landseer and absent from 170 control dogs. We observed perfect concordance of the genotypes at this variant with the muscular dystrophy phenotype in more than one thousand dogs. We have to caution here that our genetic data do not prove the causality of the identified variant. On the basis of these data we cannot formally rule out the possibility that the true causative variant is a noncoding regulatory variant or that the true causative variant was entirely missed by the chosen short read resequencing methodology in combination with an incomplete reference genome.

On the other hand, the genetic evidence provides statistical support for the causality of the *COL6A1:c.289G>T* for the observed muscular dystrophy. In addition to the genetic data, the functional knowledge on collagen type VI from other species is fully consistent with this hypothesis. *COL6A1* is actually a very good functional candidate gene for a muscular dystrophy as genetic variants in this gene cause different human myopathies with variable clinical phenotypes ranging from the milder Bethlem myopathy to the severe Ullrich congenital muscular dystrophy.

The reported variant in Landseer dogs introduces an early stop codon into the open reading frame. Although we have not experimentally analyzed the expression of the mutant *COL6A1* gene, it seems likely that the mutant transcripts will be subject to nonsense-mediated decay and that the *COL6A1:c.289G>T* variant leads to a true null allele of the *COL6A1* gene. Unfortunately, we had very limited material from the muscle biopsies and could not analyze the RNA experimentally to confirm this hypothesis.

On the basis of the combination of the available genetic data, knowledge on *COL6A1* function in other species, and the fact that the identified nonsense variant is likely to represent a full null allele, we think that the causality of the *COL6A1:c.289G>T* variant for the observed muscular dystrophy has been established beyond reasonable doubt.

In humans with collagen VI alterations, the clinical severity is variable. Ullrich’s congenital muscular dystrophy is the most severe phenotype with severe weakness and axial und proximal contractures. Affected patients lose the ability to walk within the first or second decade of life. Bethlem congenital muscular dystrophy has milder clinical signs, including moderate-to-mild muscle weakness and distal contractures only. Between Ullrich’s and Bethlem muscular dystrophies, intermediate phenotypes exist with lesser degrees of weakness and longer periods of unassisted ambulation (Bönnemann 2011). The Landseer dogs showed a severe clinical phenotype, which closely resembles human patients with Ullrich congenital muscular dystrophy. The clinical phenotype of these Landseers was more severe than that seen in a myopathic Labrador Retriever suspected to also have a defect in collagen VI (Marioni-Henry *et al.* 2014). So far, according to our knowledge, no human patients carrying two truncated *COL6A1* alleles have been reported. However, it seems plausible that homozygosity for a *COL6A1* nonsense variant in the Landseer dogs leads to a very severe phenotype with a recessive mode of inheritance. A comparable situation resulting in severe clinical phenotypes has been reported in human patients carrying truncating variants on both alleles of the *COL6A2* gene (Camacho Vanegas *et al.* 2001; Allamand *et al.* 2011).

It is interesting to note that homozygous *Col6a1^−/−^* knock out mice show a very mild phenotype that has been compared with human Bethlem myopathy although these mice do not express any residual collagen alpha1(VI) chains (Bonaldo *et al.* 1998; Irwin *et al.* 2003). This is in contrast to humans and dogs where the complete *COL6A1* deficiency leads to a much more severe phenotype. The comparatively mild murine phenotype recapitulates the findings from Duchenne muscular dystrophy: Completely *DMD*-deficient humans and dogs suffer from a very severe clinical phenotype, whereas *Dmd*-deficient mice only have a very mild phenotype (McGreevy *et al.* 2015). Thus, the Landseer dogs with the *COL6A1:c.289G>T* variant are potentially an interesting animal model for human Ullrich congenital muscular dystrophy as they resemble the severe human clinical phenotype much closer than *Col6a1^−/−^* knock out mice.

In conclusion, we provide the first comprehensive description of a clinically severe canine muscular dystrophy, most likely caused by a nonsense variant in the *COL6A1* gene. The affected dogs represent a model for human Ullrich congenital muscular dystrophy. Our findings enable genetic testing in dogs, so that the nonintentional breeding of affected Landseers can be avoided in the future.

## Supplementary Material

Supporting Information
