# Supplementary material for: A Nonsense Variant in COL6A1 in Landseer Dogs with Muscular Dystrophy
Source: G3 (Bethesda). 2015 Oct 1;5(12):2611–7. doi: 10.1534/g3.115.021923 (PMC4683634; doi:10.1534/g3.115.021923)
Supplement: Supporting Information [file supp_5_12_2611__index.html]

A Nonsense Variant in COL6A1 in Landseer Dogs with Muscular Dystrophy — Supporting Information 

# A Nonsense Variant in *COL6A1* in Landseer Dogs with Muscular Dystrophy

## Supporting Information for Steffen *et al.*, 2015

**Files in this Data Supplement:**

- Table S1 - Genome regions that showed positive LOD scores for linkage to muscular dystrophy in a family with 10 informative meioses. (.xlsx, 14 KB)
- Table S2 - Homozygous genome regions with shared alleles among the 4 analyzed cases. (.xlsx, 15 KB)
- Table S3 - *COL6A1:c.289G>T* genotypes of 473 dogs from diverse dog breeds. (.xlsx, 14 KB)
- File S1 - Clinical phenotype of a 4 month old affected Landseer. To view the video, please download the file and play it using a compatible media player. (.mp4, 7 MB)
